# Role of Flagella in the Pathogenesis of *Helicobacter pylori*

**DOI:** 10.1007/s00284-017-1256-4

**Published:** 2017-04-26

**Authors:** Haiying Gu

**Affiliations:** Medical School, Ningbo University, Ningbo, 315211 China

## Abstract

This review aimed to investigate the role of *Helicobacter pylori* flagella on the pathogenicity of this bacterium in humans. *Helicobacter pylori* is a flagellated pathogen that colonizes the human gastroduodenal mucosa and produces inflammation, and is responsible for gastrointestinal disease. Its pathogenesis is attributed to colonization and virulence factors. The primary function of *H. pylori* flagella is to provide motility. We believe that *H. pylori* flagella play an important role in the colonization of the gastrointestinal mucosa. Therefore, we reviewed previous studies on flagellar morphology and motility in order to explore the relationship between *H. pylori* flagella and pathogenicity. Further investigation is required to confirm the association between flagella and pathogenicity in *H. pylori.*

## Introduction

*Helicobacter pylori* is a flagellated microaerophilic gram-negative bacillus that is known to colonize the gastrointestinal mucosa of almost half the global human population with varying prevalence rates across different geographical regions [13]. *Helicobacter pylori* is perhaps the most infectious of all known bacteria. Although some believe that *H. pylori* is a type of “commensal bacterium” [52], it cannot be classified as normal flora because all patients with gastroduodenal *H. pylori* colonization show histological gastroenteritis [39], which can develop into a number of gastric diseases such as chronic gastritis, duodenitis, peptic ulcers (gastric and duodenal), mucosa-associated lymphoid tissue (MALT), atrophic gastritis, and gastric adenocarcinoma. This bacterium exhibits allelic diversity and genetic variability. Thus, infection might appear as a high rate of mixed infections, indicating that one person might be infected with multiple strains of *H. pylori.* The mixed infection rate is high in epidemic areas with a high incidence [35]. The pathogenesis of *H. pylori* infection is partly dependent on colonization and virulence factors [39], and flagella play an important role in the colonization of the gastrointestinal mucosa [24]. The difference (heterogeneity) in the motilities of colonizing strains was first reported by [17], but failed to attract much attention. The role of heterogeneity in the motility of *H*. *pylori* has not been sufficiently explored.

## Morphology and Structure of *H. pylori* Flagella

The bacterial flagellum is a complex motility organ composed of multiple types of protein subunits [46]. Each flagellum consists of three components [44, 53]: the basal body, hook, and filament. Electron microscopic observation of the *H. pylori* flagellum reveals the presence of a sheath and a terminal bulb [68]. The function of the membrane-like flagellar sheath of *H. pylori* is hitherto unknown, and little is known of its composition [47]. *Helicobacter pylori* has 4–8 unipolar flagella [42]; however, it remains controversial whether the flagella are uni- or bipolar [25]. Table 1 presents the structural composition and functions of *H. pylori* flagellar structures.

**Table 1:** Composition and functions of *H. pylori* flagellar structures

Structure	Composition	Function	References
Flagellar basal body	C ring (FliM, FliN, FliY, FliG)	Transfers proteins, regulates motor rotation and conversion, and coordinates protein secretion	[9, 46, 82]
	MS ring (FliF)	Involved in the synthesis of FlaA, FlaB, and FlgE	[3]
	Type III secretion system (FlhA, FliO, FlhB, FliP, FliQ, FliR)	Transports the majority of the flagellar proteins to the end of the flagellar structure	[31, 38, 77]
	Motor (MotA, MotB)	Fixes and rotates the flagellum	[16, 59]
Flagellar hook	Flagellum export chaperone (FliS)	Prevents premature polymerization of flagellin, and participates in flagellum assembly	[3, 41]
	Flagellar hook protein (FlgE)	Connects the basal body and the flagellar filament, and is closely related to the powerful driving force in a viscous environment	[14, 61, 71]
	FlgK	Controls the length of the flagellar hook during flagellum assembly	[14]
Flagellar filament	FlaA, FlaB	Plays an important role in bacterial motility	[3, 32]
	FliD	As a filament-capping protein in flagellar assembly	[36, 37]
Flagellar sheath	HpaA, FaaA	Protect against depolymerization of the flagellin subunits at low pH	[10, 21, 68]

## Functions and Pathogenicity of *H. pylori* Flagella

*Helicobacter pylori* flagella produce different types of motility [24], including “swimming motility,” “spreading motility,” and “swarming motility,” which are defined as movement in liquid media, movement in soft agar (0.3% agar concentration), and movement on the surface of semi-solid or solid media, respectively. *Helicobacter pylori* flagella may influence their colonization in bacteria, inflammation, and immune evasion.

### Colonization and Colonization sites

#### Colonization

The viability of *H. pylori* on the surface of the gastrointestinal mucosa depends on its colonization factors such as urease, motility, chemotaxis, outer membrane proteins, and the special helix morphology of the bacterium [15, 44, 53, 78, 85]. The optimal pH of *H. pylori* is neutral [28, 76], while the optimal pH of *H. pylori* in liquid media is 8.5 (slightly alkaline) [4]. Thus, the acidic environment of the stomach is not suitable for its growth. It is generally believed that urease plays a protective role in *H. pylori* [74], facilitating colonization of the mucosa of the stomach by hydrolyzing urea into NH_3_ and CO_2_ [69]. The NH_3_ produced by the action of urease neutralizes stomach acid and increases the pH of the surrounding cells [15]. In addition, urease participates in the inflammatory reaction and facilitates adhesion by interacting with the CD74 receptor on gastric epithelial cells [48, 50] isolated urease-negative *H. pylori* mutants from patients with peptic ulcers, and found that this strain successfully colonized the stomach of Mongolian gerbils and caused ulcers; therefore, the role of urease as a colonization factor is uncertain. The importance of motility as a colonization factor in *H. pylori* was first demonstrated in the study by [17], who demonstrated that germ-free piglets exhibited a higher infection rate when infected with motile *H. pylori* than when infected with the non-motile strain; moreover, the motile strain also colonized the stomachs for a longer duration in the stomach of germ-free piglets. Similarly, many animal studies using motility-deficient mutants, including the *H. pylori motB* mutant [62], *fliD* mutant [37], *putA* mutant [56], and a chemotaxis mutant strain [49, 56], have shown similar findings. The spreading motility of these mutant strains is weak and their colonization in the stomach of the animals was reduced. The most convincing evidence for the role of motility in *H. pylori* colonization came from the study by [5, 60] found that changes in flagellin glycosylation affected strain motility. When the glycosylation level of the FlaA protein was increased, the strain motility and colonization load both increased. These studies have convincingly indicated that flagellar motility is an important factor influencing colonization. The colonization of *H. pylori* is expressed as colonization density or load. The number of colonies per gram of gastric mucosa (CFU/g) [6, 49] is determined by quantitative culture. *Helicobacter pylori* DNA can be quantitatively measured by polymerase chain reaction (PCR) [20].

Motility is measured by three different methods. First, swimming motility can be directly determined by the average swimming velocity of bacteria in the gastric mucosal layer using phase-contrast microscopy [8]. Second, spreading motility can be determined by assessing the growth ring diameter in semi-solid agar using puncture inoculation [49, 60, 62]. Third, swarming motility can be determined by examining the growth ring diameter on the surface of semi-solid medium [51, 56] using quantitative inoculation. These studies showed that the *H. pylori* colonization load in gastric mucosa was positively correlated with motility in animal infection models.

#### Colonization Site

Colonization by *H. pylori* is not evenly distributed. Colonization in the stomach is usually observed at the gastric antrum [51]. However, *H. pylori* colonies can also be found in other sites. For example, *H. pylori* has been known to colonize the duodenum and is recognized as the primary cause of idiopathic duodenal ulcers [65, 66]. Although the incidence is low (6.9%), it can be assumed that the strains colonizing different gastrointestinal sites might have different origins, because *H. pylori* infection in the stomach has been demonstrated to be heterogeneous [35]. *Helicobacter pylori* may also colonize the colon [57, 58, 81], but its origin remains unclear.

### Immune Inflammation and Evasion

The motility and colonization load of *H. pylori* are positively correlated with neutrophilin filtration [1, 34, 49]. Colonization is the basis of the inflammatory reaction induced by *H. pylori*, and motility is a critical colonization determinant that affects the infection outcome. Furthermore, the flagellum also influences inflammation and immune evasion.

#### Immune Inflammation

The main structural proteins of the *H. pylori* flagellum include HpaA, FlaA, FlaB, FliD, and FlgK. Of these, HpaA, FlaA, and FlaB have been found to be expressed in *H. pylori* strains isolated from biopsy specimens of patients with stomach disease. These flagellins are the primary targets of the humoral immunity after infection, and induce antibody responses [79]. In contrast, an important study showed that FlaA was antigenic but not immunogenic [72]. The role of *H. pylori* flagellins in immune inflammation is yet unknown.

#### Immune Evasion

*Helicobacter pylori* infections usually occur during childhood and last for a lifetime if left untreated with antibiotics [45]. The human immune system cannot eliminate these bacteria primarily because of the bacterial ability of immune evasion. Although *H. pylori* flagellin can induce anti-flagellin antibodies in infected patients, it is not recognized by toll-like receptor 5 (TLR5), a member of the toll-like receptor family, which is activated by most bacterial flagellins [1, 54]. One possible reason may be that the flagellins, especially FlaA, are not exposed, and thus cannot be detected in the infected gastric epithelial cells [22]. Other bacterial flagella induce interleukin 8 (IL-8) secretion, leading to an inflammatory reaction. However, *H. pylori* flagellin does not typically induce IL-8 secretion in gastric epithelial cells. Although highly motile strains of *H. pylori* have been shown to elicit a higher level of IL-8 production [42], the flagellar sheath HpaA probably shields the flagellin from recognition by TLR5 [10].

## Relationship Between *H. pylori* Flagellar Structure, Motility, Chemotaxis, and Colonization

In the flagellar structure, the C ring complex is composed of FliM, FliN, FliY, and FliG. Typically, *fliM*, *fliY*, and *fliG* mutant *H. pylori* strains cannot produce flagella. Although the *fliN* mutant strain can produce flagella, they are “paralyzed” and unable to move [46], resulting in a non-motile bacterium. In the flagellar structure, the “motor” is important for bacterial motility. *motB*-deficient *H. pylori* have been reported to exhibit normal flagellar structure but no motility, and the colonization load of this strain is significantly lower than controls containing *motB* in infected mice [62]. *fliF*, *fliS*, *flhB*, *fliQ*, *fliG*, or *fliI* mutant strains did not produce any flagella and were non-motile, while the *flhA* mutant strain produced short flagella [3]. FlgE is the main protein of the flagellar hook, and strains lacking the *flgE* gene expectedly showed no motility [61]. FlaA and FlaB are the components of the flagellar filament and are important for motility. Strains lacking the *flaA* and *flaB* genes exhibit reduced irregular flagella and lower motility. The *flaA* and *flaB* double-mutant strain is completely non-motile. The *flaA* and *flaB* mutant strains have reduced colonizing ability [18, 32], and cannot colonize even with a longer period of incubation in animal models [18]. Mutation of the flagellar filament *fliD* gene results in non-motile bacteria with short flagella, and this strain is unable to colonize the gastric mucosa of mice [37]. The FaaA protein is required for flagellar and proper flagellar localization as well as for optimal flagellar function. This protein is exported to the outer membrane and subsequently becomes a component of the flagellar sheath. *Helicobacter  pylori* mutant strains deficient in *faaA* exhibited decreased motility and less efficient colonization of the stomach in mice compared to the wild-type *H. pylori* strain at the early stages of infection [10, 68].

Flagellar hook substructure reaches its optimal length sensed by the ‘checkpoint control’ protein FliK, export of the anti-sigma factor FlgM is triggered releasing σ^28^ from a σ^28^-FlgM complex which in turn allows the subsequent expression of σ^28^ dependent genes. In *fliK,* mutants hook to filament transition do not occur and long hooks of unregulated length termed polyhooks are formed [55]. It is demonstrated that FliK is necessary for upregulation of *cagA.* Expression and flagellar regulatory system of *H. pylori* is directly required for upregulation of the major virulence gene *cagA* in gastric cell associated *H. pylori.*

The direction of flagellar rotation is cooperatively controlled by the chemotaxis-signaling protein CheY and the flagellar rotor protein FliN [44]. Strains with mutant chemotaxis genes *cheW*, *cheV* [67], *cheY*, *cheA* [8], and *tlpB* [49] have less motility and reduced colonization load [49, 80]. In contrast, a study by Williams et al. demonstrated that chemotaxis gene mutants (ΔcheY, ΔcheW) displayed an adequate colonization load but a reduced inflammatory response [83]. Reference [30] investigated a new protein, ChePep, located in the flagellar pole, which regulates flagellar rotation and controls *H. pylori* chemotaxis. Strains with the *ChePep* gene mutation exhibited reduced flagellar motility.

The only known phosphatase in *H. pylori* is CheZ, called CheZHP in this system. It is reported that CheZHP localization depends on the ChePep chemotaxis protein [29] and conversely ChePep localization depends on CheZHP, which raises the intriguing possibility that some phosphatases, including CheZHP and ChePep, exist in a complex that is distinct from the core chemotaxis signaling and flagellar complexes [43].

Reference [70] demonstrated that the colonization of a TlpD-controlled chemotaxis gene mutant strain in the gastric antrum was significantly reduced. Similarly, it was found that the colonization of strains with chemotaxis gene mutants (Δ*cheY*) and especially the motility gene mutants (Δ*motB*) was reduced [1]. Future studies should clarify the relationship between chemotaxis and colonization.

All members of the *Epsilonproteobacteria* have their flagella located at either one or both cellular poles [2, 27, 40, 64, 73, 75, 84]. *Campylobacter jejuni* and *Helicobacter pylori* are the most studied epsilonproteobacteria because they are important human pathogens. In addition to their unique structural features revealed by cryoelectron tomography [11], *Campylobacter* and *Helicobacter* flagella exhibit unique aspects in the regulation of the expression of their flagellar genes and in the assembly of their flagellar structure [23, 44]. Regulation of flagellar gene expression in *Campylobacter* and *Helicobacter* is also unique, involving a two-component system (FlgRS), the FlhF GTPase, and the transcription factors σ^54^ and σ^28^ [7, 33, 63].

## Discussion

Although many components of the *H. pylori* lagella have been characterized and data regarding flagellar function and regulation are rapidly increasing, certain aspects of the *H. pylori* system, in particular those that differ from the well-studied model systems, are still poorly understood and require further investigation. These regulatory mechanisms appear to act at the bottom of the putative transcriptional hierarchy that governs flagellar biosynthesis in *H. pylori*. In contrast, the mechanisms at the top of the hierarchy that actually trigger the initiation of flagellar gene transcription are completely unknown.

Previous studies have explored the spreading and swarming motilities; however, whether these two types of motility are equivalent remains unclear.

Adhesion is an important factor that mediates the pathogenic role of bacterial flagellum [19, 26]. However, unlike other bacteria, *H. pylori* adhesion on gastric epithelial cells is not dependent on flagellin [12] and is not influenced by reduction in bacterial flagella. Moreover, it is related to mutations of flagellar genes. For example, the adhesion ability of a *flaA::cat*/*flab::km* mutant strain without flagella is adequate while that of the *flbA* mutant strain is significantly reduced.

Exploring the relationship between *H. pylori* flagellar motility and gastrointestinal mucosa colonization can facilitate the understanding of *H. pylori* pathogenesis, especially the heterogeneity of motility in mixed infections, and needs to be further investigated.
